# Lancemaside A from *Codonopsis lanceolata* Modulates the Inflammatory Responses Mediated by Monocytes and Macrophages

**DOI:** 10.1155/2014/405158

**Published:** 2014-03-23

**Authors:** Eunji Kim, Woo Seok Yang, Ji Hye Kim, Jae Gwang Park, Han Gyung Kim, Jaeyoung Ko, Yong Deog Hong, Ho Sik Rho, Song Seok Shin, Gi-Ho Sung, Jae Youl Cho

**Affiliations:** ^1^Department of Genetic Engineering, Sungkyunkwan University, Suwon 440-746, Republic of Korea; ^2^Medical Beauty Research Institute, AmorePacific R&D Center, Yongin 446-729, Republic of Korea; ^3^Department of Herbal Crop Research, National Institute of Horticultural & Herbal Science, Rural Development Administration, Eumseong 369-873, Republic of Korea

## Abstract

In this study, we aimed to examine the cellular and molecular mechanisms of lancemaside A from *Codonopsis lanceolata* (Campanulaceae) in the inflammatory responses of monocytes (U937 cells) and macrophages (RAW264.7 cells). Lancemaside A significantly suppressed the inflammatory functions of lipopolysaccharide- (LPS-) treated RAW264.7 cells by suppressing the production of nitric oxide (NO), the expression of the NO-producing enzyme inducible NO synthase (iNOS), the upregulation of the costimulatory molecule CD80, and the morphological changes induced by LPS exposure. In addition, lancemaside A diminished the phagocytic activity of RAW264.7 cells and boosted the neutralizing capacity of these cells when treated with the radical generator sodium nitroprusside (SNP). Interestingly, lancemaside A strongly blocked the adhesion activity of RAW264.7 cells to plastic culture plates, inhibited the cell-cell and cell-fibronectin (FN) adhesion of U937 cells that was triggered by treatment with an anti-*β*1-integrin (CD29) antibody and immobilized FN, respectively. By evaluating the activation of various intracellular signaling pathways and the levels of related nuclear transcription factors, lancemaside A was found to block the activation of inhibitor of *κ*B kinase (IKK) and p65/nuclear factor- (NF-) *κ*B. Taken together, our findings strongly suggest that the anti-inflammatory function of lancemaside A is the result of its strong antioxidative and IKK/NF-*κ*B inhibitory activities.

## 1. Introduction

Inflammation is a body defense system that protects us from exogenous pathogens, such as bacteria, viruses, and fungi [1, 2]. To recognize these pathogens, toll-like receptors (TLRs) play a critical role in delivering information on the state of infection to the cell so that it can initiate a host protection response [3]. Macrophages and monocytes are the major cells of the immune system and express high levels of TLRs on their cell membranes [4]. The activation of TLRs in macrophages and monocytes triggers numerous cellular and molecular responses. Morphological changes as well as increases in phagocytic uptake, migration, and adhesion are the primary responses of these cells to exogenous pathogens [5]. Following these responses, the cells produce various inflammatory mediators, such as nitric oxide (NO) and prostaglandin E_2_ (PGE_2_), and proinflammatory cytokines, such as tumor necrosis factor- (TNF-) *α*, interleukin- (IL-) 1, IL-6, and interferon- (IFN-) *β*, and increase the expression of surface molecules, including costimulatory molecules, such as CD80 and CD86, and adhesion molecules, such as *β*1-integrin (CD29), by activating intracellular signaling cascades, including the phosphatidylinositide 3-kinase (PI3 K), AKT, inhibitor of *κ*B (I*κ*B) kinase (IKK), and I*κ*B*α* pathways, and the subsequent activation of transcription factors, such as nuclear factor- (NF-) *κ*B, via the upregulation of the cellular redox system [1, 2, 6–8]. As chronically sustained inflammatory responses are understood to cause numerous serious diseases, such as cancer, diabetes, atherosclerosis, and Alzheimer's disease, various methods to suppress inflammatory responses could be good therapeutic approaches to prevent or cure these diseases [9–13].

**Figure 1:**
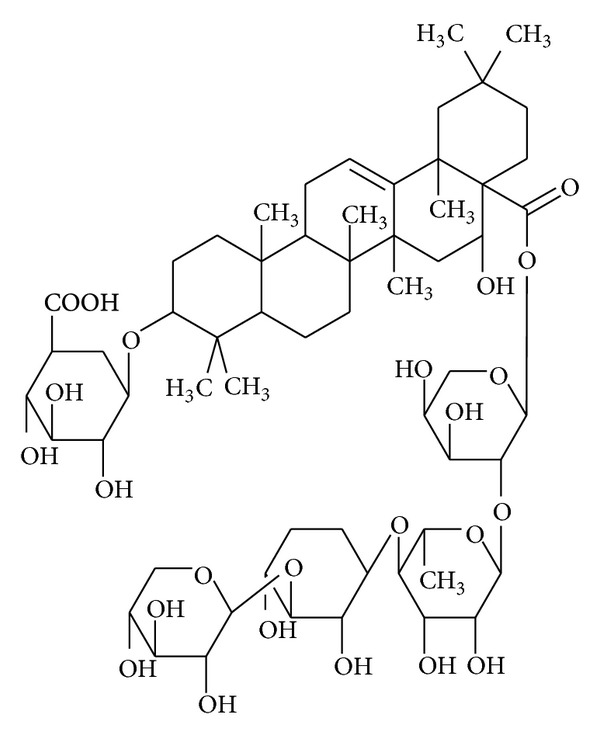
Chemical structure of lancemaside A.

*Codonopsis lanceolata* (family Campanulaceae) is a popular medicinal herb that is used to prevent various lung inflammations, such as bronchitis and cough [14]. In addition, this plant has been demonstrated to exhibit antidiabetic, anticancer, antiobesity, antilipogenic, and hepatoprotective activities [15–18]. Lancemaside A (Figure 1) is a representative triterpenoid saponin that highlights the pharmacological activities of* Codonopsis lanceolata* [19, 20]. In fact, it was previously reported that this saponin is able to suppress scopolamine-induced memory and learning deficits in mice [19] and to ameliorate 2,4,6-trinitrobenzenesulfonic acid-induced colitis [21]. To understand the anti-inflammatory activities of* Codonopsis lanceolata* and its major ingredient, lancemaside A, its cellular and molecular mechanisms have been under study. Thus, the methanol extract of* Codonopsis lanceolata* roots and its saponin-rich subfraction have been revealed to suppress the functional activation of macrophages [22, 23]. Of the many saponin components, lancemaside A was demonstrated to suppress the activation of the TLR-linked NF-*κ*B and its upstream kinase interleukin-1 receptor-associated kinase 4 (IRAK-4) in the peritoneal macrophages and intestines of mice [21, 24].

Although recent studies on* Codonopsis lanceolata* roots, the saponin-rich subfraction, and lancemaside A have expanded our understanding of their anti-inflammatory mechanism, the exact targets of the NF-*κ*B inhibitory pathway and the other cellular target pathways that are modulated by the single saponin component lancemaside A in macrophages and monocytes have not yet been fully elucidated. Therefore, in the present study, the mechanisms of lancemaside A were carefully explored in terms of its molecular and cellular targets in macrophage/monocyte-mediated inflammatory responses.

## 2. Materials and Methods

### 2.1. Materials

Sodium nitroprusside (SNP), 3-(4,5-dimethylthiazol-2-yl)-2,5-diphenyltetrazolium bromide (MTT), dihydrorhodamine 123 (DHR123), Fluorescein isothiocyanate- (FITC-) dextran, and lipopolysaccharide (LPS;* E. coli* 0111:B4) were purchased from Sigma Chemical Co. (St. Louis, MO, USA). BAY11-7082 (BAY) was obtained from Calbiochem (La Jolla, CA, USA). Fibronectin (FN), fetal bovine serum, and RPMI 1640 were obtained from Gibco (Grand Island, NY, USA). The murine macrophage cell line RAW264.7 and the human promonocytic cell line U937 were purchased from the American Type Culture Collection (ATCC) (Rockville, MD, USA). The aggregation-inducing anti-CD29 antibody (MEM 101A) was used as reported previously [25]. The FITC-conjugated anti-CD80 antibody was from PharMingen (San Diego, CA, USA). All other chemicals were of analytical grade and were obtained from Sigma. The phosphospecific and/or total antibodies against intercellular adhesion molecule 1 (ICAM-1), p65, the activation protein 1 (AP-1), family proteins c-Fos and c-Jun, I*κ*B*α*, IKK, AKT, extracellular signal-regulated kinases (ERK), c-Jun N-terminal kinase (JNK), and *β*-actin were obtained from Cell Signaling (Beverly, MA, USA).

### 2.2. Preparation of Lancemaside A

Lancemaside A was isolated from* Codonopsis lanceolata*, which was obtained from Hoengseong (Kangwon-Do, Korea), according to previous reports [14, 20]. The root of the plant was used in this study. The dried root (1 kg) was extracted with 70% MeOH and then divided into three aliquots that were mixed with petroleum ether, EtOAc, or* n*-BuOH. The* n*-BuOH extract was fractionated by HP-20 resin elution using a water-MeOH solvent gradient. The active fraction (MeOH fraction) was further separated by MPLC (a dichloromethane-MeOH solvent gradient), and then lancemaside A (130 mg) was isolated. This compound exhibited more than 97% purity in HPLC analysis.

### 2.3. Cell Culture

RAW264.7 and U937 cells were cultured in RPMI 1640 medium supplemented with 10% heat-inactivated fetal bovine serum (FBS; Gibco, Grand Island, NY, USA), glutamine, and antibiotics (penicillin and streptomycin) at 37°C under 5% CO_2_. For each experiment, the cells were detached with a cell scraper. When the cells were cultured for the experiments at 2 × 10^6^ cells/mL, the proportion of dead cells was less than 1% as determined by Trypan blue dye exclusion.

### 2.4. NO Production

RAW264.7 macrophage cells (1 × 10^6^ cells/mL) were cultured for 18 h, pretreated with lancemaside A (0 to 30 *μ*M) for 30 min, and further incubated with LPS (1 *μ*g/mL) for 24 h. To check whether lancemaside A can directly inhibit NO release, lancemaside A was incubated with SNP (10 mM) in a microtube for 30 min. The inhibitory effect of lancemaside A on LPS-induced or SNP-derived NO production was determined by analyzing the NO level using Griess reagent as previously described [26, 27]. The OD at 550 nm (OD_550_) was measured using a SpectraMax 250 microplate reader (Molecular Devices, Sunnyvale, CA, USA).

### 2.5. Cell Viability Test

RAW264.7 and U937 cells (1 × 10^6^ cells/mL) were cultured for 18 h, after which lancemaside A (0 to 50 *μ*M) was added to the cells for the final 24 or 8 h of culture. The cytotoxic effect of lancemaside A was then evaluated by a conventional MTT assay as reported previously [28, 29]. For the final 3 h of culture, 10 *μ*L MTT solution (10 mg/mL in phosphate buffered saline, pH 7.4) was added to each well. The incubation was halted by the addition of 15% sodium dodecyl sulfate (SDS) to each well, which solubilized the formazan [30]. The absorbance at 570 nm (OD_570–630_) was measured using a SpectraMax 250 microplate reader (BioTek, Bad Friedrichshall, Germany).

### 2.6. Analysis of iNOS Expression by Real-Time Reverse Transcription Polymerase Chain Reaction

RAW264.7 cells (1 × 10^6^ cells/mL) were cultured for 18 h, pretreated with lancemaside A (0 to 40 *μ*M) for 30 min, and further cultured with LPS (1 *μ*g/mL) for 6 h. The inhibitory effect of lancemaside A on the expression of iNOS was determined by real-time quantitative RT-PCR [26, 31]. To determine the iNOS gene expression level, total RNA was isolated from LPS-treated RAW264.7 cells using TRIzol Reagent (Gibco BRL) according to the manufacturer's instructions. The total RNA was stored at −70°C until use. Real-time quantitative (q) RT-PCR was conducted as reported previously [32, 33]. The primers (Bioneer, Daejeon, Korea) used in these reactions are listed in Table 1.

### 2.7. Flow Cytometric Analysis

The expression of CD80 in RAW264.7 cells that were treated with lancemaside A for 30 min and then stimulated with LPS (1 *μ*g/mL) for 8 h was determined by flow cytometric analysis as reported previously [25]. The stained cells were analyzed on a FACScan flow cytometer (Becton-Dickinson, San Jose, CA, USA).

### 2.8. Morphological Analysis

RAW264.7 cells were pretreated with lancemaside A for 30 min and then incubated with LPS for 12 h. Images of these cells in culture at the indicated time points were obtained using an inverted phase contrast microscope that was interfaced with a video camera and NIH image software.

### 2.9. Determination of Phagocytic Uptake

To measure the phagocytic activity of RAW264.7 cells, we modified a previously reported method [34]. RAW264.7 cells (5 × 10^4^) that were pretreated with lancemaside A or BAY11-7082 for 1 h were resuspended in 100 *μ*L PBS containing 1% human AB serum and incubated with FITC-dextran (1 mg/mL) at 37°C for 20 min. The reactions were stopped by adding 2 mL of ice-cold PBS containing 1% human serum and 0.02% sodium azide. The cells were then washed three times with cold PBS-azide and analyzed on a FACScan flow cytometer as reported previously [35].

### 2.10. Determination of Reactive Oxygen Species Generation

The level of intracellular ROS was determined by recording the change in fluorescence that resulted from the oxidation of the fluorescent probe DHR123. Briefly, 5 × 10^5^ RAW264.7 cells were exposed to lancemaside A or BAY11-7082 for 30 min and then incubated with SNP (0.25 mM) at 37°C for 20 min to induce ROS production. The cells were further incubated with 20 *μ*M of the fluorescent probe DHR123 for 30 min at 37°C. The degree of fluorescence, which corresponded to the level of intracellular ROS, was determined using a FACScan flow cytometer (Becton-Dickinson, San Jose, CA, USA) as reported previously [35].

### 2.11. Cell Adhesion Assay

To test the effect of lancemaside A on the plastic adherence of cells, a wound healing assay was performed as reported previously [36]. Monolayers of RAW264.7 cells were grown to 90% confluence in 6-well plates. A wound was created by scraping each monolayer with a P200 pipette tip [37]. The wounded monolayer was washed three times with PBS to remove any cellular debris, and lancemaside A was added to the cells for 30 min. The wounds were then photographed with a digital camera, and the free cells in wound area were counted with a cell counter.

A U937 cell-cell adhesion assay was performed as reported previously [25, 38]. Briefly, U937 cells were preincubated with lancemaside A for 30 min and then further incubated with the function-activating (agonistic) anti-CD29 antibody (1 *μ*g/mL) in a 96-well plate. After a 3 h incubation, the cultures were examined with an inverted light microscope equipped with a COHU high-performance CCD (Diavert) video camera. Four random fields of each well were captured and analyzed using NIH image software to calculate the average size of the clusters. For the cell-fibronectin adhesion assay, U937 cells (5 × 10^5^ cells/well) pretreated with lancemaside A or BAY11-7082 were seeded on a fibronectin (50 *μ*g/mL) coated plate and incubated for 4 h [39]. After removing the unbound cells with PBS, the attached cells were treated with 0.1% of crystal violet for 15 min. The OD at 570 nm was measured with a SpectraMax 250 microplate reader.

### 2.12. Preparation of Cell Lysates and Immunoblotting Analysis

RAW264.7 or U937 cells (5 × 10^6^ cells/mL) were washed three times with cold PBS with 1 mM sodium orthovanadate, resuspended in lysis buffer (20 mM Tris-HCl, pH 7.4, 2 mM EDTA, 2 mM ethyleneglycoltetraacetic acid, 50 mM *β*-glycerophosphate, 1 mM sodium orthovanadate, 1 mM dithiothreitol, 1% Triton X-100, 10% glycerol, 10 *μ*g/mL aprotinin, 10 *μ*g/mL pepstatin, 1 mM benzamidine, and 2 mM PMSF), lysed by sonication, and rotated for 30 min at 4°C. The lysates were clarified by centrifugation at 16,000 ×g for 10 min at 4°C and stored at −20°C until use. The soluble fractions of the cell lysates were immunoblotted, and the total and phosphoprotein levels of ICAM-1, p65, c-Jun, c-Fos, I*κ*B*α*, IKK, AKT, ERK, JNK, and *β*-actin were visualized as previously reported [40].

### 2.13. IKK*α* and IKK*β* Kinase Assay

To evaluate the ability of lancemaside A to inhibit the activity of purified IKK*α* and IKK*β*, we used the Millipore Kinase Profiler service (Billerica, MA, USA) as reported previously [41]. Human IKK*α* or IKK*β* (1–5 mU) was incubated in reaction buffer in a final reaction volume of 25 *μ*L. The reaction was initiated by the addition of MgATP. After incubation for 40 min at room temperature, the reaction was stopped by the addition of 5 mL of a 3% phosphoric acid solution. Ten microliters of the reaction product was spotted onto a P30 filtermat and washed three times for 5 min each with 75 mM phosphoric acid and once in methanol prior to drying and scintillation counting.

### 2.14. Statistical Analysis

Data are expressed as the mean ± standard deviation (SD), as calculated from one (*n* = 6) of two independent experiments. Other data are representative of three different experiments with similar results. For statistical comparisons, the results were analyzed using analysis of variance/Scheffe's post hoc test and the Kruskal-Wallis/Mann-Whitney test. A *P*  value <0.05 was considered to be statistically significant. All statistical tests were conducted using SPSS (SPSS Inc., Chicago, IL, USA).

## 3. Results and Discussion

Lancemaside A, one of the major triterpenoid saponins isolated from* Codonopsis lanceolata* (Campanulaceae), is traditionally prescribed for treating various lung inflammatory diseases, including bronchitis and cough. As the anti-inflammatory mechanism of lancemaside A has not been fully elucidated, in this study, we aimed to examine the cellular and molecular events that occur in response to lancemaside A treatment in the inflammatory responses of monocytes (U937 cells) and macrophages (RAW264.7 cells).

**Figure 2:**
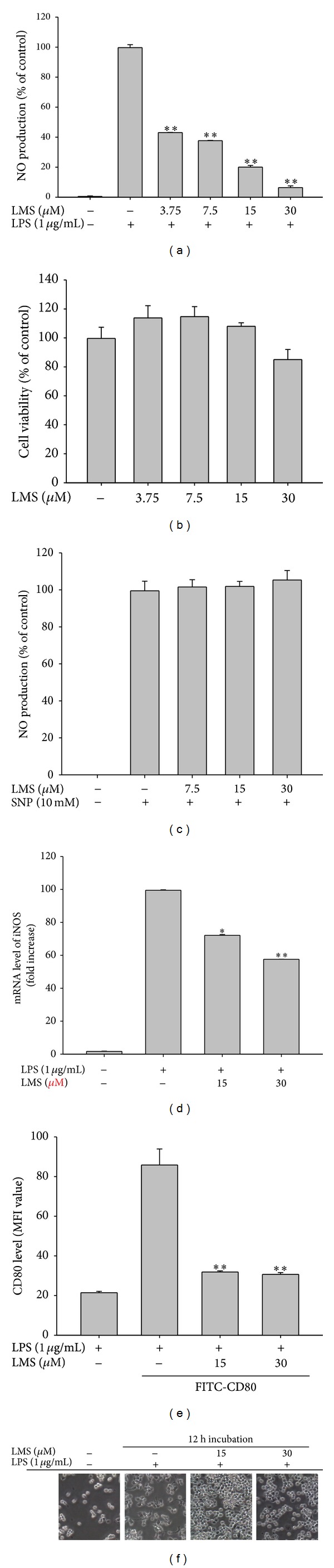
Effect of lancemaside A on the activation of RAW264.7 cells during LPS exposure and on the viability of RAW264.7 cells. ((a) and (c)) The NO level in the culture supernatant of RAW264.7 cells treated with LPS for 24 h (a) or with SNP for 20 min (b) was analyzed by Griess assay. (c) The viability of RAW264.7 cells treated with lancemaside A was determined by MTT assay. (d) The level of iNOS mRNA in RAW264.7 cells treated with lancemaside A (0 to 30 *μ*M) in the presence or absence of LPS (1 *μ*g/mL) for 6 h was determined by real-time quantitative RT-PCR. (e) The surface level of CD80 in RAW264.7 cells treated with LPS for 12 h was determined by flow cytometric analysis. (f) Images of the cells in culture at 12 h were obtained with an inverted phase contrast microscope that was interfaced with a video camera and NIH image software. MFI: mean fluorescence intensity; *P < 0.05 and **P < 0.01 versus control.

**Figure 3:**
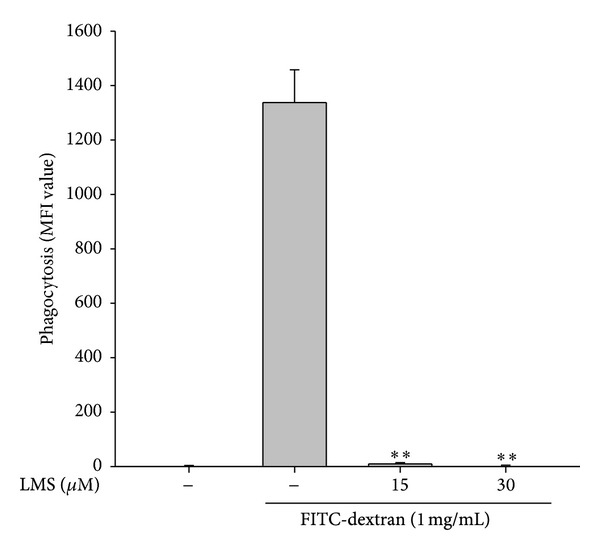
Effect of lancemaside A on phagocytic uptake in RAW264.7 cells. RAW264.7 cells were preincubated with lancemaside A and then treated with FITC-dextran (1 mg/mL) for 20 min. The level of dextran uptake was determined by flow cytometric analysis. **P < 0.01 versus control.

**Figure 4:**
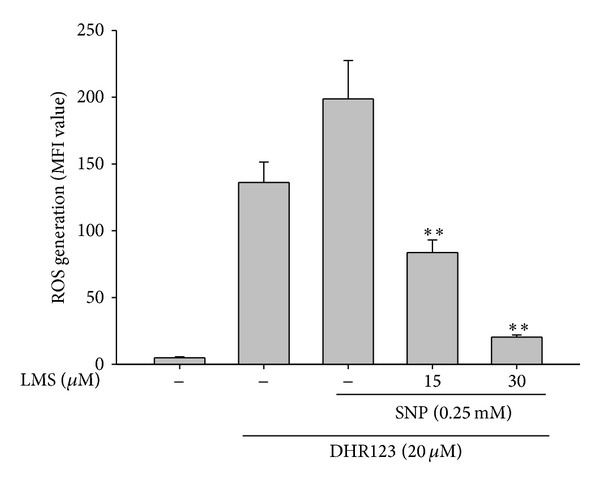
Effect of lancemaside A on reactive oxygen species (ROS) generation in sodium nitroprusside- (SNP-) treated RAW264.7 cells. RAW264.7 cells that were preincubated with lancemaside A were treated with DHR123 (20 *μ*M) in the presence or absence of SNP (0.25 mM) for 20 min. The level of intracellular ROS was determined by flow cytometric analysis. MFI: mean fluorescence intensity. **P < 0.01 versus control.

We first examined whether lancemaside A is able to modulate the functional activation of macrophages upon TLR4 stimulation. To do this, we treated RAW264.7 cells with LPS, a TLR4 ligand, to trigger macrophage-mediated inflammatory responses. In fact, LPS treatment enhanced NO production, increased iNOS expression, upregulated the surface level of CD80, and boosted morphological changes in the RAW264.7 cells (Figure 2). Like the methanol extract and saponin subfraction of* Codonopsis lanceolata* [22, 23], lancemaside A strongly inhibited NO production up to 90% (Figure 2(a)) without altering the cell viability (Figure 2(b)). Considering that lancemaside A did not neutralize SNP-induced NO production (Figure 2(c)), it is expected that the inhibitory activity of lancemaside A on NO production was not due to the direct scavenging activity of NO but occurred in response to the suppression of the NO production pathway. Indeed, this compound strongly suppressed the mRNA expression of the NO-releasing enzyme iNOS (Figure 2(d)), also implying that lancemaside A affects the inflammatory signaling pathways that contribute to transcriptional regulation. In agreement with this finding, lancemaside A blocked the upregulation of surface CD80, a costimulatory molecule that aids in the interaction between macrophages and T cells [42], which was stimulated by LPS exposure (Figure 2(e)). Interestingly, this compound completely suppressed the morphological alteration of RAW264.7 cells that is triggered by LPS (Figure 2(f)), indicating that the regulatory pathways that induce this morphological change could also be targeted by this compound. Similarly, the phagocytic uptake of FITC-dextran, which requires morphological and cytoskeletal changes [43], was strongly inhibited by lancemaside A up to 95% at 15 *μ*M (Figure 3). Moreover, this compound suppressed the ROS generation induced by SNP in a dose-dependent manner in RAW264.7 cells (Figure 4). The finding that lancemaside A did not directly suppress the SNP-induced release of NO radicals (Figure 2(c)) strongly implies that lancemaside A does not act as a strong antioxidative compound but rather acts as a positive regulator of the cellular antioxidative system. Because there are no reports on the functional involvement of lancemaside A in the upregulation of the cellular redox system, we will carefully examine this possibility in future experiments.

**Figure 5:**
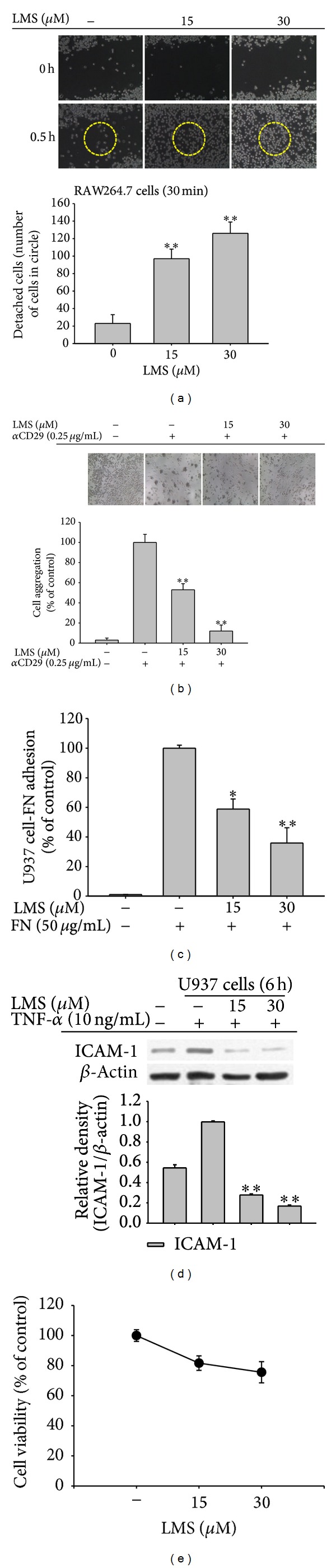
Effects of lancemaside A on RAW264.7 and U937 cell adhesion. (a) Effect of lancemaside A on the plastic adherence of RAW264.7 cells was analyzed using a wound healing assay. After scratching and washing the cells, lancemaside A was added for 30 min. The extent of detachment was determined by counting the free cells in the circle containing the wound area. Images of the cells were obtained using an inverted phase contrast microscope that was interfaced with a video camera and NIH image software. (b) U937 cells (1 × 10^6^ cells/mL) that were pretreated with lancemaside A were incubated in the presence or absence of the aggregation-inducing anti-CD29 antibody (0.25 *μ*g/mL) for 3 h. Images of the cells in culture were obtained using an inverted phase contrast microscope that was attached to a video camera. Quantitative evaluation of the U937 cell-cell clusters was performed by counting the cells in each cluster. (c) Effect of lancemaside A on cell-fibronectin (FN) adhesion. U937 cells were pretreated with lancemaside A and seeded on FN (50 *μ*g/mL) coated plates for 4 h. The number of attached cells was determined by crystal violet staining. (d) The level of ICAM-1 in TNF-*α*-treated U937 cells was examined by immunoblotting analysis of whole cell lysates. (e) The viability of RAW264.7 cells treated with lancemaside A was determined by MTT assay. *P < 0.05 and **P < 0.01 versus normal or control.

The most important finding of this study is that lancemaside A is capable of modulating the adhesion of RAW264.7 and U937 cells. When RAW264.7 cells were treated with lancemaside A, we found that this compound strongly blocked the plastic adherence of these cells. To verify this pharmacological feature, we employed a wound healing assay in which we scratched a monolayer of RAW264.7 cells, washed away the free cells, and then added lancemaside A. As shown in Figure 5(a), a 30 min incubation with this compound was sufficient to detach and disperse the cells in the scratched area. As it is known that the adherence of cells to plastic culture flasks is dependent on the functional activation of adhesion molecules [44], we next employed an assay in which adhesion was triggered by the activation of CD29, a major adhesion molecule that regulates the cell-cell or cell-matrix protein adhesion of macrophages and monocytes [45, 46]. As shown in Figure 5(b), the treatment of U937 cells with an agonistic anti-CD29 antibody, which activated CD29, stimulated cell-cell aggregation, whereas lancemaside A suppressed the formation of U937 cell clusters in a dose-dependent manner, implying that CD29-mediated cell-cell adhesion is pharmacologically modulated by this compound. Indeed, our group has found that several chemicals, including ceramide, cynaropicrin, cinnamaldehyde, and ginsenoside Rp1, act as negative regulators of the CD29-mediated homotypic aggregation of U937 cells [7, 46–48], while staurosporine, 20S-dihydroprotopanaxatriol, and PMA act as positive modulators, increasing the clustering of U937 cells [49, 50]. To confirm the inhibitory activity of lancemaside A on CD29 activation, U937 cell-matrix protein adhesion was induced with immobilized fibronectin, a ligand of CD29. As shown in Figure 5(c), this compound diminished U937 cell-fibronectin adhesion in a dose-dependent manner, implying that the functional activation of *β*1-integrin is negatively targeted by lancemaside A.

In addition, the stimulation of monocytes by cytokines, such as TNF-*α*, is known to boost the adhesion of monocytes to the endothelial cell layer in blood vessels. During this event, the most critical step is the upregulation of adhesion molecules (e.g., ICAM-1) in monocytes [51]. Fortunately, an experimental protocol using U937 cells and recombinant TNF-*α* to mimic this event has been already published [52], and, using this protocol, many scientists have screened for novel anti-inflammatory drugs that target the adhesion of monocytes to blood vessel endothelial cells [52–54]. Under our conditions, we clearly found that lancemaside A was able to completely suppress the expression of the ICAM-1 protein in U937 cells (Figure 5(d)) without altering cell viability (Figure 5(e)). Therefore, these results strongly indicate that lancemaside A modulates the adherence of macrophages and monocytes, either between cells or between cells and matrix proteins.

**Figure 6:**
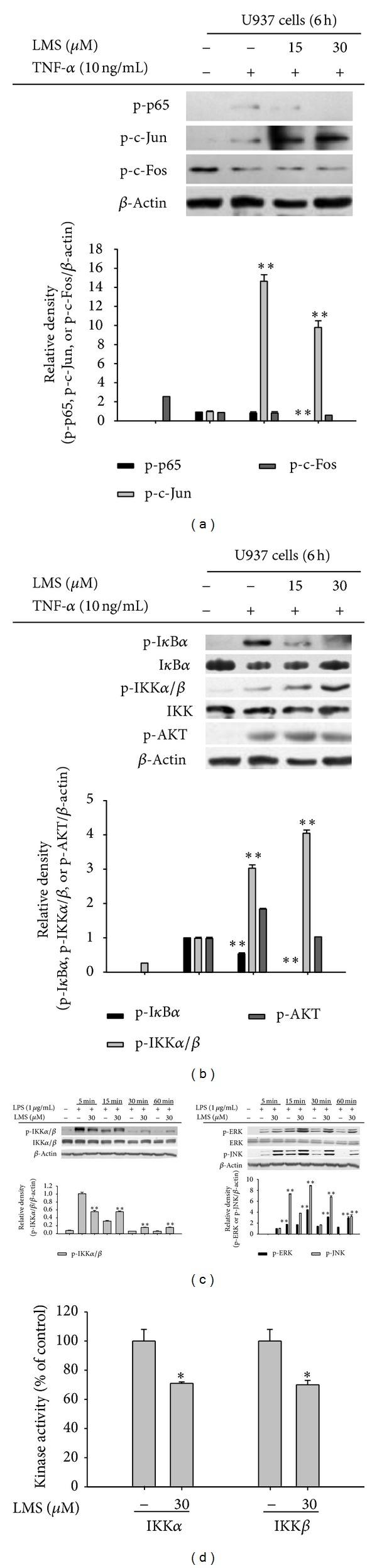
Effect of lancemaside A on the activation of transcription factors and their upstream signaling cascades. (a) The phosphoprotein levels of p65, c-Jun, and c-Fos in the whole cell lysates of TNF-*α*-treated U937 cells were determined by immunoblotting analysis. ((b) and (c)) The phosphoprotein and total protein levels of I*κ*B*α*, IKK, AKT, ERK, JNK, and *β*-actin from the cell lysates were determined by immunoblotting analysis. (d) The kinase activities of IKK*α* and IKK*β* were determined by a direct kinase assay using purified enzymes. The value of the control, which received vehicle treatment, was set as 100% activity for each enzyme (IKK*α* or IKK*β*). *P < 0.05 versus control.

**Figure 7:**
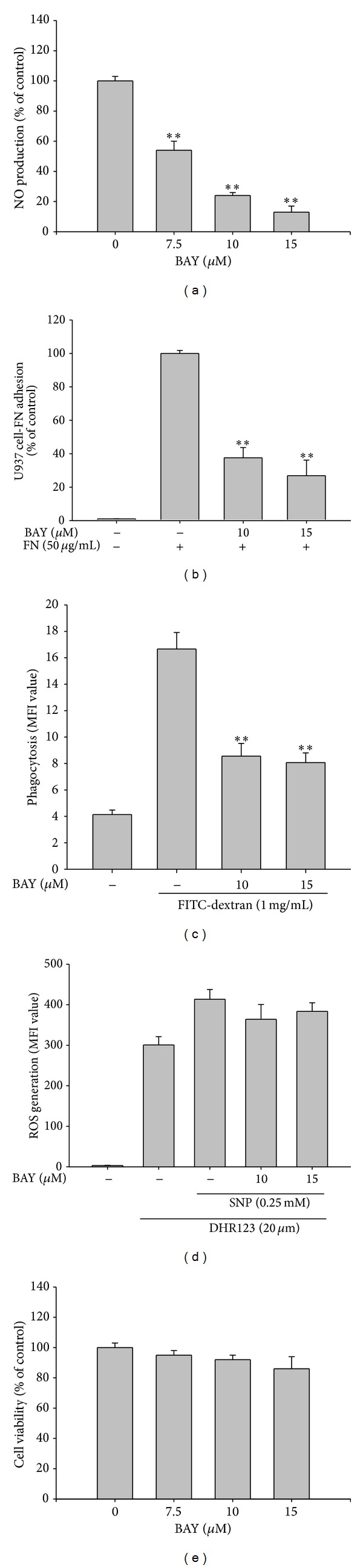
Effect of BAY11-7082 (BAY) on the inflammatory responses of U937 and RAW264.7 cells. (a) The NO level in the culture supernatant of RAW264.7 cells treated with LPS for 24 h was analyzed by Griess assay. (b) The effect of BAY11-7082 on cell-fibronectin (FN) adhesion was examined with U937 cells that were pretreated with BAY11-7082 and seeded on FN (50 *μ*g/mL) coated plates for 4 h. The number of attached cells was determined by crystal violet staining. (c) RAW264.7 cells were preincubated with BAY11-7082 and then treated with FITC-dextran (1 mg/mL) for 20 min. The level of dextran uptake was determined by flow cytometric analysis. (d) RAW264.7 cells were preincubated with BAY11-7082 and then treated with DHR123 (20 *μ*M) in the presence or absence of SNP (0.25 mM) for 20 min. The level of intracellular ROS was determined by flow cytometric analysis. (e) The viability of RAW264.7 cells treated with BAY11-7082 was determined by MTT assay. MFI: mean fluorescence intensity. **P < 0.01 versus control.

Considering that lancemaside A blocked the expression of iNOS mRNA in LPS-treated RAW264.7 cells (Figure 2(d)) and the expression of the ICAM-1 protein in TNF-*α*-treated U937 cells (Figure 5(d)), it was obvious to next test whether this compound can block the activation of transcription factors. Indeed, previously, the inhibition of NF-*κ*B by lancemaside A was observed in LPS-treated RAW264.7 cells, TLR4-expressing HEK293 cells, and 2,4,6-trinitrobenzenesulfonic acid-treated colons [21, 24], although the exact inhibitory targets of this compound were not identified. As expected, in the present study, lancemaside A suppressed the phosphorylation of p65 (Figure 6(a)), a subunit of NF-*κ*B, which is a critical process for NF-*κ*B activation in TNF-*α*-treated U937 cells [55]. Unlike the finding that lancemaside A suppresses AP-1 activation in LPS-stimulated BV2 microglial cells [56], unfortunately, we did not observe the inhibition of AP-1 (c-Jun and c-Fos) under our conditions, which was most likely due to the use of different incubation times and stimuli. Further analysis of the upstream signaling pathway that contributes to NF-*κ*B activation led us to uncover that lancemaside A suppresses I*κ*B*α* phosphorylation without inhibiting IKK*α*/*β* phosphorylation in TNF-*α*-treated U937 cells at 5 min to 6 h (Figure 6(b) and Figure 6(c) left panel). In contrast, no inhibition of ERK and JNK phosphorylation was observed (Figure 6(c) right panel). Thus, this result implies that IKK*α*/*β* acts as a direct target enzyme in the pharmacological action of lancemaside A. To address this assumption, we employed a direct kinase assay with purified IKK*α* and IKK*β* proteins. Although the inhibitory potency of lancemaside A was not drastic, the results shown in Figure 6(d) strongly indicated that lancemaside A significantly suppressed the kinase activity of IKK*α*/*β* up to 60%. From this simple test to confirm the identity of the inhibitory pathway that regulates NF-*κ*B activation in LPS-treated peritoneal macrophages in this system (via analysis of IRAK4 expression, IKK*β* phosphorylation, and I*κ*B*α* phosphorylation), we identified IKK*α*/*β* as a direct target of lancemaside A, expanding our knowledge on the molecular pharmacological action of lancemaside A. Moreover, these IKKs were verified as lancemaside A target proteins using the IKK inhibitor BAY11-7082 (BAY). BAY exhibited similar inhibitory activities to lancemaside A on NO production in LPS-treated RAW264.7 cells (Figure 7(a)), cell-FN adhesion in U937 cells (Figure 7(b)), and phagocytic uptake in FITC-dextran-treated RAW264.7 cells (Figure 7(c)), without suppressing cell viability in U937 cells (Figure 7(e)). However, BAY did not suppress SNP-induced radical generation, indicating that the indirect radical scavenging activity of lancemaside A is not regulated by its IKK/NF-*κ*B inhibitory action. Because ROS and RNS, which are generated upon macrophage activation, have been reported to stimulate IKK/NF-*κ*B activation, unlike BAY11-7082, the neutralizing activity of lancemaside A can be added to its list of NF-*κ*B inhibitory pathway functions.

**Figure 8:**
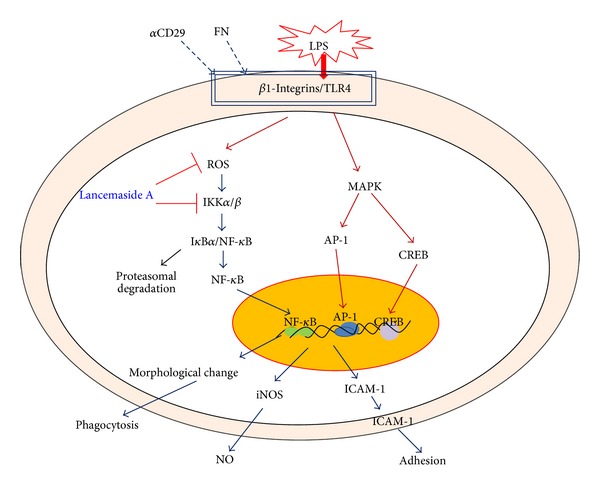


In summary, lancemaside A was able to suppress various inflammatory responses that are managed by macrophages and monocytes. This compound inhibited the release of NO, the expression of iNOS, the upregulation of CD80, the induction of morphological changes, and the increases in phagocytic activity, ROS generation, and cell-cell and cell-FN adhesion by suppressing the activity of IKK and p65/NF-*κ*B, as summarized in Figure 8. Taken together, our findings strongly suggest that the anti-inflammatory mechanism of lancemaside A involves the suppression of the cellular responses of macrophages and monocytes by blocking redox activation and the IKK/NF-*κ*B pathway. Based on our data and previous reports, we also propose that lancemaside A can be further developed as the first anti-inflammatory drug prepared from* Codonopsis lanceolata*. Therefore, our future studies will focus on examining this possibility.

## Figures and Tables

**Table 1:** Primer sequences used in the RT-PCR analysis.

Name		Sequence (5′ to 3′)
iNOS	F	CCCTTCCGAAGTTTCTGGCAGCAG
	R	GGCTGTCAGAGCCTCGTGGCTTTGG

COX-2	F	CACTACATCCTGACCCACTT
	R	ATGCTCCTGCTTGAGTATGT

TNF-*α*	F	TGCCTATGTCTCAGCCTCTTC
	R	GAGGCCATTTGGGAACTTCT

IFN-*β*	F	TCCAAGAAAGGACGAACATTCG
	R	GAGGCCATTTGGGAACTTCT

GAPDH	F	CACTCACGGCAAATTCAACGGCA
	R	GACTCCACGACATACTCAGCAC
